# Ligandability Assessment of Human Glutathione Transferase M1-1 Using Pesticides as Chemical Probes

**DOI:** 10.3390/ijms23073606

**Published:** 2022-03-25

**Authors:** Charoutioun S. Bodourian, Nirmal Poudel, Anastassios C. Papageorgiou, Mariana Antoniadi, Nikolaos D. Georgakis, Hiroshi Abe, Nikolaos E. Labrou

**Affiliations:** 1 Laboratory of Enzyme Technology, Department of Biotechnology, School of Applied Biology and Biotechnology, Agricultural University of Athens, 75 Iera Odos Street, 118 55 Athina, Greece; haris.bod@gmail.com (C.S.B.); mariana10antoniadi@gmail.com (M.A.); n.georgakis@aua.gr (N.D.G.); 2 Turku Bioscience Centre, University of Turku and Åbo Akademi University, 20521 Turku, Finland; nirpou@utu.fi (N.P.); anapap@utu.fi (A.C.P.); 3 Department of Chemistry, Graduate School of Science, Nagoya University, Furo-cho, Chikusa-Ku, Nagoya 464-8602, Japan; abe.hiroshi@c.mbox.nagoya-u.ac.jp

**Keywords:** CDNB, 1-chloro-2,4-dinitrobenzene, GSH, glutathione, GST, glutathione transferase, G-site, GSH binding site, H-site, hydrophobic binding site, GS-ESF, S-glutathionyl ethylenesulfonyl fluoride

## Abstract

Glutathione transferases (GSTs; EC 2.5.1.18) form a group of multifunctional enzymes that are involved in phase II of the cellular detoxification mechanism and are associated with increased susceptibility to cancer development and resistance to anticancer drugs. The present study aims to evaluate the ligandability of the human GSTM1-1 isoenzyme (hGSTM1-1) using a broad range of structurally diverse pesticides as probes. The results revealed that hGSTM1-1, compared to other classes of GSTs, displays limited ligandability and ligand-binding promiscuity, as revealed by kinetic inhibition studies. Among all tested pesticides, the carbamate insecticide pirimicarb was identified as the strongest inhibitor towards hGSTM1-1. Kinetic inhibition analysis showed that pirimicarb behaved as a mixed-type inhibitor toward glutathione (GSH) and 1-chloro-2,4-dinitrobenzene (CDNB). To shine a light on the restricted hGSTM1-1 ligand-binding promiscuity, the ligand-free crystal structure of hGSTM1-1 was determined by X-ray crystallography at 1.59 Å-resolution. Comparative analysis of ligand-free structure with the available ligand-bound structures allowed for the study of the enzyme’s plasticity and the induced-fit mechanism operated by hGSTM1-1. The results revealed important structural features of the H-site that contribute to xenobiotic-ligand binding and specificity. It was concluded that hGSTM1-1 interacts preferentially with one-ring aromatic compounds that bind at a discrete site which partially overlaps with the xenobiotic substrate binding site (H-site). The results of the study form a basis for the rational design of new drugs targeting hGSTM1-1.

## 1. Introduction

Glutathione transferases (GSTs, EC 2.5.1.18) comprise a family of widely distributed phase II detoxification enzymes that catalyze the conjugation of a broad variety of reactive electrophiles to the nucleophilic sulfur atom of the intracellular thiol, tripeptide glutathione (γ-L-Glu-L-Cys-Gly) [1]. Glutathione transferases typically serve as a crucial host-defense mechanism, acting as scavengers of electrophilic xenobiotics and their reactive metabolites [2].

Four major families of proteins that are widely distributed in nature exhibit glutathione transferase activity. Two of these, the cytosolic and mitochondrial GSTs, comprise soluble enzymes. The third family comprises microsomal GSTs and is now referred to as the membrane-associated proteins in eicosanoid and glutathione metabolism (MAPEG). The fourth family of GSTs is represented by the bacterial fosfomycin-resistance proteins FosA and FosB. The human cytosolic GSTs constitute the largest family and are divided into seven classes based on sequence similarity: alpha, mu, pi, theta, zeta, omega and sigma [3,4]. The tertiary structure of cytosolic GSTs consists of two subunits, homodimeric or heterodimeric. Each subunit consists of 200–250 amino acids with a molecular weight of 25–30 kDa and an active center [5]. Each subunit contains an active site that consists of a GSH-binding site (G-site) in the N-terminal domain and a site that binds the hydrophobic substrate (H-site) in the C-terminal domain [6]. The H-site shows low homology between isoenzymes within each class and amongst different classes and can bind with a large variety of substrates which are different in size, structure, and hydrophobicity. Therefore, the structure of the H-site determines substrate specificity for different xenobiotic compounds [7,8,9]. Additionally, GSTs act as ligand-binding proteins where numerous hydrophobic and amphipathic compounds can bind. This non-catalytic site is called the L-site and its exact operation has not yet been determined [8,10].

Glutathione transferases comprise a family of enzymes, well-known for their multiple functions but mainly involved in the detoxification mechanism of various xenobiotic electrophile molecules. Among the electrophile molecules that are detoxified are chemotherapeutic agents, which are used in the treatment of different types of cancer. It is well-known that certain GSTs classes, namely a, μ and π, are involved in the development of anti-cancer drug-resistance phenomena [11]. For example, GSTA1-1 and GSTP1-1 were reported to be, in short, connected with an increased risk of gastric, breast and pancreatic cancer [1,12,13,14,15,16]. Moreover, the isoenzyme GSTM1-1 is mostly studied in humans for its correlation with many types of cancer [13,14,15], while other studies support its pertinence with Parkinson’s disease [17,18]. Many studies have shown that an overexpression of GSTM1-1 may have direct impact on chemoresistance [13,19]. This is achieved either through immediate detoxification or by inhibiting the action of the MAP kinase pathway [12,20]. Several GSTs are involved in protein–protein interactions with mitogen-activated protein kinases (MAPKs), modulating apoptosis pathways and cellular proliferation. Interestingly, in silico and in vitro studies have shown that hGSTM1-1 interacts with high binding affinity to MAPK8 [21], although the catalytic activity of hGSTM1-1 is not directly affected [22]. The functional significance of GSTs and their involvement in diverse cellular processes, including conferring resistance to chemotherapy, justify the need for research efforts aiming at the discovery of new inhibitors targeting GSTs [23]. For example, the potent inhibitor, 6-(7-nitro-2,1,3-benzoxadiazol-4-ylthio)hexanol (NBDHEX) showed high inhibition potency towards both GSTP1 and GSTM1 [16]. Furthermore, natural compounds such as curcumin and the flavonoid fisetin display high inhibitory potency towards human GSTA1 and GSTP1, respectively [24,25]. Georgakis et al. [19], have synthesized and studied a series of 2,2’-dihydroxybenzophenone analogues that showed high inhibition potency towards hGSTM1-1. Another class of GST inhibitors are the glutathionyl-analogues (i.e., GS-conjugates) [26]. For example, the S-glutathionyl ethylenesulfonyl fluoride (GS-ESF) is a GST irreversible inhibitor that affects the function of several GSTs [27,28]. GS-ESF is a GSH derivative, in which the –SH group has been substituted by the electrophilic reactive group sulfonyl fluoride, allowing for the covalent reaction of GS-ESF with several GSTs [28].

In the present work, we screened the inhibition potency of a wide range of different pesticides towards hGSTM1-1 in order to assess whether registered chemical scaffolds (e.g., pesticides), with well-established chemical and toxicological profiles, can provide new lead compounds for further drug-design research. Given the material costs and the slow rate of new drug discovery and design, repurposing of existed chemical scaffolds is becoming a very attractive proposition, because in that way, the drug-development timeline is dramatically reduced since various existing compounds have already-known technology, safety and toxicity profiles [29]. The results of the present work allowed for the mapping of the xenobiotic ligand-binding properties of hGSTM1-1 and provided structure/function relationships that can be further exploited as a basis for the design of new and selective lead compounds as inhibitors towards hGSTM1-1.

## 2. Results and Discussion

### 2.1. Ligandability Assessment of hGSTM1-1 by Inhibition Analysis

**Figure 1:**
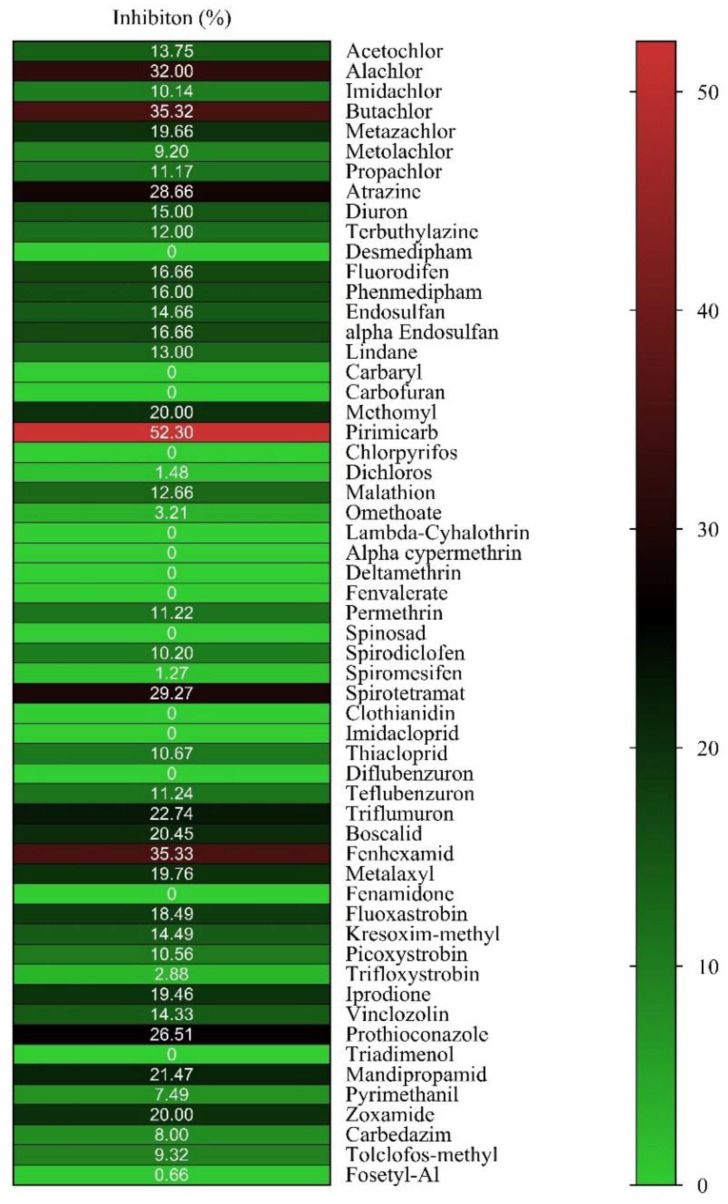
Screening of inhibition potency of pesticides (25 μM) towards hGSTM1-1. The isoenzyme was assayed in all measurements using CDNB-GSH assay system. The colors represent the mean values of three inhibition assays (%) for each pesticide against the tested enzyme with a variation less than 5% in all cases.

In the present work, the ligandability of hGSTM1-1 was assessed using a library of pesticides that belong to different chemical families, including insecticides, herbicides, and fungicides. The members of the library were selected to represent a diverse group of compounds with different structures and physicochemical properties. The provided structural diversity allows for the mapping of the enzyme’s ligand-binding site and the characterization of the enzyme’s binding specificity. The inhibition sensitivity of hGSTM1-1 towards each member of the library was initially assessed at a final concentration of 25 μM and the results are illustrated in Figure 1.

**Figure 2:**
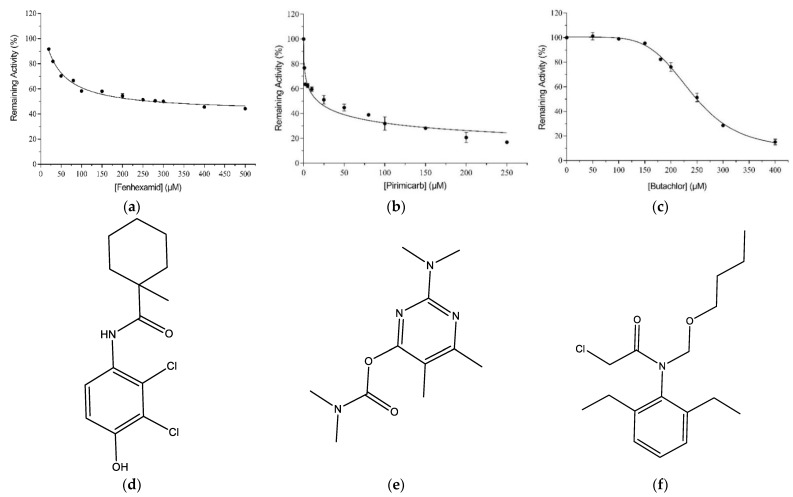
Concentration–response curve for fenhexamid (**a**), pirimicarb (**b**), and butachlor (**c**). IC_50_ for hGSTM1-1 was determined by fitting Equation (4) to the data through non-linear curve-fitting methods. The structures of fenhexamid, pirimicarb, and butachlor are shown in (**d**), (**e**) and (**f**), respectively.

From the results presented in Figure 1, it is evident that the hGSTM1-1 exhibits restricted ligandability compared to other GSTs that belong to different classes (e.g., alpha, pi, tau) [19,30,31]. Among all tested pesticides, only three compounds, namely pirimicarb, fenhexamid, and butachlor, displayed higher inhibition potency and were selected for IC_50_ determination (Figure 2). Although pirimicarb appears to exhibit higher inhibition potency, we attempted to measure the IC_50_ values for the three inhibitors for validating the results of the initial screening experiment (Figure 1).

The IC_50_ values of pirimicarb, fenhexamid, and butachlor at 37 °C and pH = 6.5 were 17.51 ± 1.75 μM (R^2^ = 0.96), 277.9 ± 14.5 μM (R^2^ = 0.98), and 236.3 ± 4.5 μM (R^2^ = 0.99), respectively. From the IC_50_ values and data from earlier studies [19,31,32], it appears that fenhexamid and butachlor fall outside the expected range for a high-affinity ligand, whereas the IC_50_ value for pirimicarb is within the range expected for a promising lead compound, and therefore, pirimicarb was selected for further study.

### 2.2. Kinetic Inhibition Study of the hGSTM1-1 with the Insecticide Pirimicarb

**Figure 3:**
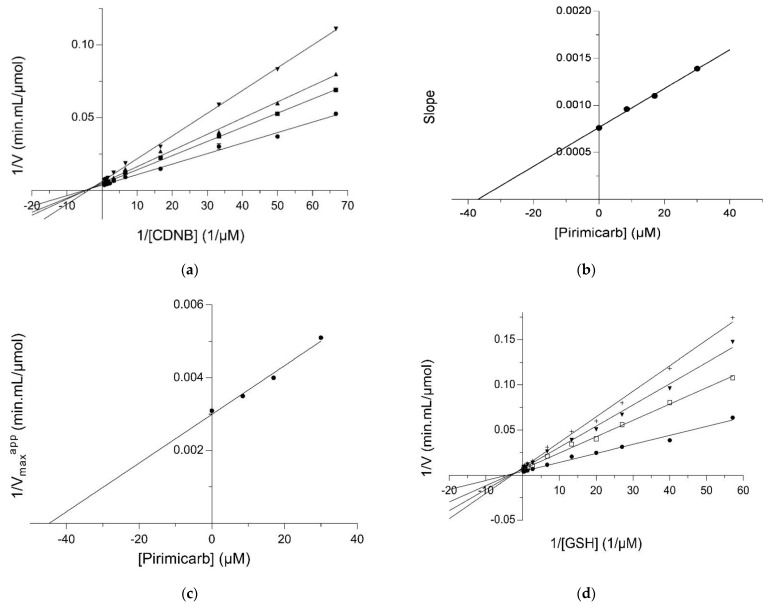

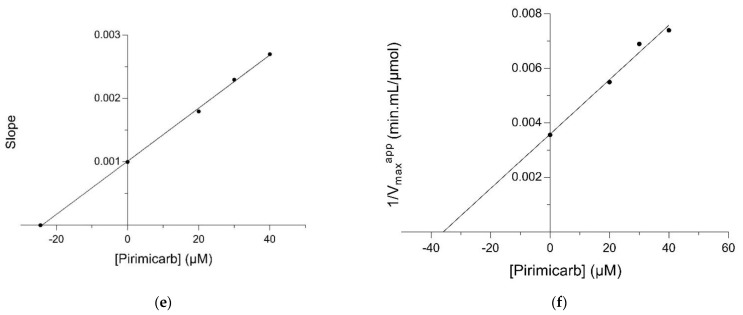
Kinetic inhibition study. (**a**): Lineweaver–Burk plot for the inhibition of hGSTM1-1 with pirimicarb at different CDNB concentrations. Pirimicarb concentrations: 0 μM, (●); 8.5 μM, (■); 17 μM, (▲); 30 μM, (▼); (**b**): Secondary (derivative) diagram. The data obtained from the Lineweaver–Burk diagram. The point of intersection with the *x*-axis corresponds to the K_i_ inhibitory constant; (**c**): Secondary (derivative) diagram. The data obtained from the Lineweaver–Burk diagram. The point of intersection with the *x*-axis corresponds to the K_i_’ inhibitory constant; (**d**): Lineweaver–Burk plot for the inhibition of hGSTM1-1 by pirimicarb at different GSH concentrations. 0 μM, (●); 20 μM, (□); 30 μM, (▼); 40 μM, (†); (**e**): Secondary (derivative) diagram. The data were obtained from the Lineweaver–Burk diagram. The point of intersection with the *x*-axis corresponds to the K_i_ inhibitory constant; (**f**): Secondary (derivative) diagram. The data obtained from the Lineweaver–Burk diagram. The point of intersection with the *x*-axis corresponds to the K_i_’ inhibitory constant.

Kinetic inhibition analysis was accomplished aiming to localize the pirimicarb binding site on hGSTM1-1. The inhibition patterns are illustrated in Figure 3. Pirimicarb exhibited a mixed-type inhibition pattern towards CDNB, as the lines of the Lineweaver–Burk plot (Figure 3a) are not intersecting the CDNB concentration axes. The observed mixed-type inhibition pattern suggests that the inhibitor can bind to either the free enzyme (inhibition constant: K_i_) or the enzyme–CDNB complex (inhibition constant: K_i_’), but their affinities for these two forms of the enzyme are different. The inhibition constants, K_i_ and K_i_’, were calculated from linear secondary graphs, depicting Slope versus [Pirimicarb] (Figure 3b) and 1/V_max_^app^ versus [Pirimicarb] (Figure 3c), and were found to be equal to 37.69 ± 1.03 μM, (R^2^ = 0.99) and 44.7 ± 1.14 μM, (R^2^ = 0.98), respectively (K_i_ < K_i_’). Similarly, using GSH as a variable substrate, pirimicarb exhibited a mixed-type inhibition, as the lines of the Lineweaver–Burk plot are not intersecting the GSH concentration axes. The mixed-type inhibition observed indicates that the inhibitor pirimicarb binds to both the free enzyme and the enzyme–GSH complex, but their affinities for these two forms of the enzyme are different. Based on the secondary graphs, the inhibitory constant K_i_ and K_i_’ were calculated as 24.47 ± 2.86 μM, (R^2^ = 0.98, Figure 3e) and 35.97 ± 2.57 μM, (R^2^ = 0.98, (Figure 3f), respectively (K_i_ < K_i_’).

### 2.3. Irreversible Inactivation of hGSTM1-1 by S-Glutathionyl Ethylenesulfonyl Fluoride (GS-ESF)

**Figure 4:**
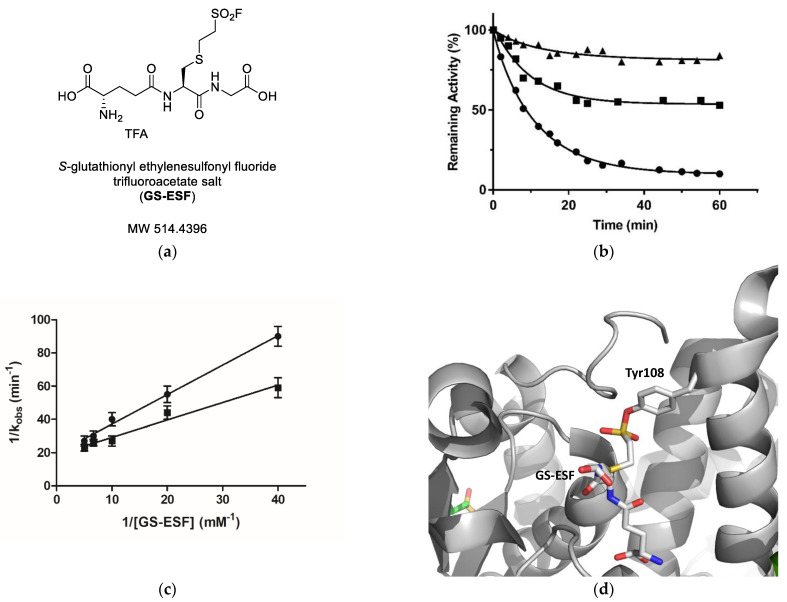
(**a**): The structure of GS-ESF [41]. (**b**): Time course of the hGSTM1-1 by GS-ESF at pH 7.4 and 25 °C, and 0.2 mM GS-ESF (●); 0.2 mM GS-ESF in the presence of S-nitrobenzyl-GSH (1 mM, ■); 0.2 mM GS-ESF in the presence of pirimicarb (20 μM, ▲). (**c**): Effect of GS-ESF concentration on the observed rate of inactivation (k_obs_) of hGSTM1-1 by GS-ESF expressed as a double reciprocal. Inactivation was carried out in the presence (●) or absence (■) of pirimicarb. The slope and intercept of the secondary double-reciprocal plot were calculated by unweighted linear regression analysis. (**d**): Crystal structure of GS-ESF bound to *h*GSTP1-1 (PDB ID code 5x79) [41]. The GSTP1-1 (gray) is shown as a cartoon and the adduct formed by GS-ESF and Tyr108 is shown in stick representation and colored according to atom type.

GS-ESF (Figure 4a) is a GST irreversible (covalent) inhibitor that inhibits the functions of several GSTs. GS-ESF is a GSH derivative, in which the -SH group was substituted by the electrophilic reactive group sulfonyl fluoride, allowing for the covalent reaction of GS-ESF with a nucleophile group of the H-site of GSTs. For example, GS-ESF specifically reacts with the H-site residue Tyr108 of GSTP1-1 [28].

Incubation of hGSTM1-1 in the presence of GS-ESF displays a time-dependent loss in catalytic activity, as expected for an irreversible inhibitor (Figure 4b). Furthermore, the catalytic activity of hGSTM1-1 cannot be regained by extensive dialysis or gel filtration on Sephadex G-25. Linearity in semilogarithmic plots of the irreversible inactivation process is observed with GS-ESF concentrations 0.025–0.2 mM. According to Kitz and Wilson kinetics [33], the following equation describes the reaction between an active site-directed reagent (e.g., GS-ESF) and an enzyme (E) [34,35,36,37]:

1/k_obs_ = 1/k_3_ + K_D_/(k_3_ [GS-ESF]) (1)

where k_obs_ is the observed rate of enzyme inactivation for a given concentration of GS-ESF, k_3_ is the maximal rate of inactivation (min^−1^), [GS-ESF] is the concentration of S-glutathionyl ethylenesulfonyl fluoride and K_D_ is the apparent dissociation constant of the hGSTM1-1 and GS-ESF complex. A plot of 1/k_obs_ versus 1/[GS-ESF] for several concentrations of GS-ESF yields a straight line with a positive ordinate intercept (k3) of 0.052 ± 0.02 min^−1^ and a slope corresponding to an apparent dissociation constant of 0.055 ± 0.01 mM (Figure 4c). These results are consistent with the Kitz and Wilson kinetics [34] that GS-ESF forms a Michaelis-type reversible complex with hGSTM1-1, and the formation of the covalent product is rate-limiting.

Incubation of hGSTM1-1 in the presence of GS-ESF and the inhibitors S-nitrobenzyl-GSH or pirimicarb diminish the observed rate of enzyme inactivation (k_obs_) and behaves as a competitive inhibitor versus GS-ESF, as shown in Figure 4b. The following kinetic equation describes the inactivation in the presence of competing ligands (e.g., pirimicarb):

1/k_obs_ = 1/k_3_ + K_D_/(k_3_ [GS-ESF])(1 + ([Pirimicarb]/K_app_)) (2)

where [Pirimicarb] is the concentration of pirimicarb and K_app_ its apparent dissociation constant of Pirimicarb. The apparent dissociation constant of pirimicarb (Figure 4c) was calculated (K_app_ = 15.5 ± 1.2 μM) from a double-reciprocal plot of the apparent rate constants (k_obs_) of hGSTM1-1 inactivation versus GS-ESF concentration, in the presence of pirimicarb. The K_app_ calculated by inactivation experiments is in good agreement to that determined by kinetic inhibition analysis.

Previous investigations have established that GS-ESF specifically reacts with the H-site residue Tyr108 of GSTP1-1 [18] (Figure 4d). Since the structurally equivalent residue in hGSTM1-1 is Tyr115, it is conceivable to propose that the same residue is also the target for GS-ESF in the case of hGSTM1-1. Tyr115 is an exposed to the solvent residue, located at the upper part of α-Helix 4, and accessible to chemical modification by GS-ESF. Tyr115 is one of the main structural determinants of the H-site [19,38,39]. All these results further support the outcome of the kinetics inhibition studies and point to the conclusion that the binding-site of pirimicarb is located at a specific position in the H-site. Of note, Hearne and Colman (2006) have found that Tyr115 contributes to the formation of the monobromobimane (mBBr) binding site, which is localized to an area midway through α-helix 4 (residues 90–114) [40].

### 2.4. The Crystal Structure of hGSTM1-1

Over the course of the project, the crystal structure of the enzyme was determined at 1.59 Å resolution. As the crystal structure of ligand-free hGSTM1-1 has been previously reported at lower (2.68 Å) resolution (PDB id 1gtu, [24]), the new structure here allows for a more detailed description of the structural features and better definition of atoms’ positions as well as the detailed investigation of the induced-fit mechanism of ligand binding and catalysis. The high-resolution structure reported here includes 1149 water atoms compared to 64 in the low-resolution hGSTM1-1 structure (PDB id 1gtu, [38]).

The enzyme was crystallized in the *P*2_1_ space group resulting in four molecules (A, B, C, D) in the asymmetric unit. In the low-resolution hGSTM1-1 structure (PDB id 1gtu), the enzyme was also crystallized with four molecules in the asymmetric unit but in a different space group (*P*2_1_2_1_2_1_). The arrangement of the molecules is similar in both structures. Each of the molecules consists of 218 residues. D–A and C–B form the typical dimers found in GSTs. The interface area of D–A and C–B dimers is 1341.9 Å^2^ and 1313.1 Å^2^, respectively. Both dimers also display a significant number of hydrogen bonds and salt bridges between them. The D–A interface has 13 H-bonds and 11 salt bridges while the C–B interface possesses 12 H-bonds and 13 salt bridges. In 1gtu, the dimer interface (~1351 Å^2^) is similar to that reported here, but the number of hydrogen bonds and salt bridges is lower, possibly owing to the low resolution of the structure that affects the accurate positioning of the atoms. Other interfaces display comparatively smaller interface areas, for example, the interface areas between B and D and C and A are 375 Å^2^ and 350 Å^2^, respectively, with only two salt bridges in both interfaces and seven and six H-bonds between them, respectively. The interactions between dimers are mediated through helix α7 (residues 178–189).

**Figure 5:**
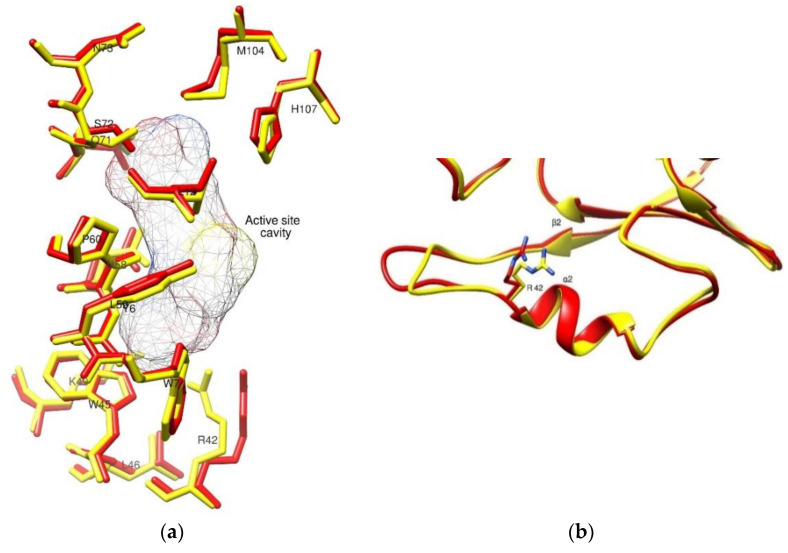
(**a**): Superposition of the active site residues of high-resolution (1.59 Å) structure of human GSTM1-1 (colored yellow) and low-resolution (2.68 Å) structure of HGSTM1-1 (colored red) (PDB id 1gtu). The active site location is denoted by a mesh surface created for GSH. (**b**): Close-up view of the loop region connecting β2 strand and α2 helix.

The structural alignment showed subtle changes among the active site residues in the low- and high-resolution hGSTM1 structures (Figure 5). The most noticeable difference was observed in Arg42, which is involved in the formation of a salt bridge with GSH and points closer to the active site cavity in the high-resolution hGSTM1 structure than in its low-resolution counterpart (Figure 5a). A small difference in the loop region connecting the β2 strand and the α2 helix is also present between the two structures. The Root-Mean-Square Deviation (RMSD) value for this region is 1.36 Å, which is higher than the RMSD value for the overall structure (0.65 Å). Furthermore, slight differences in the conformations of His107, Met104, and Ser72 were also observed (Figure 5b).

### 2.5. Prediction of the Pirimicarb Binding Site by Molecular Docking

**Figure 6:**
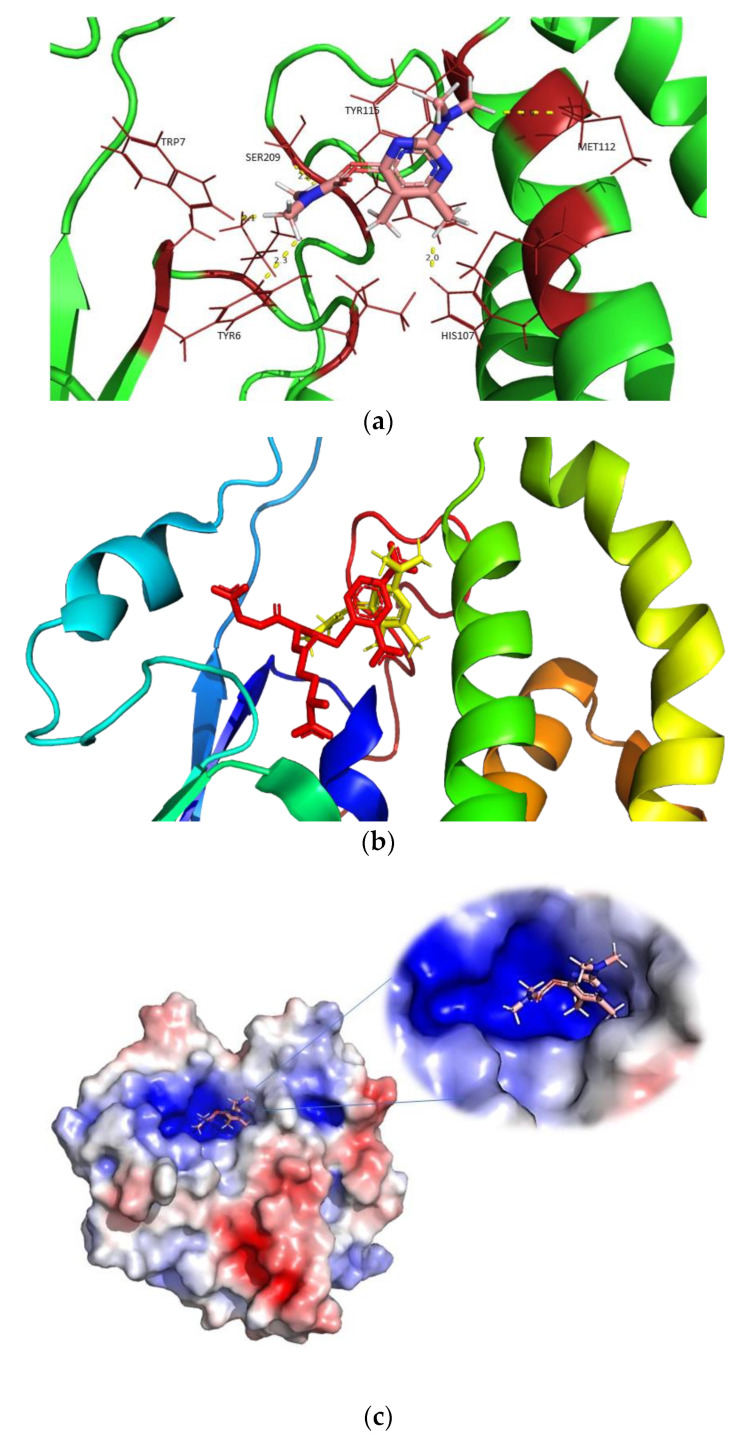
(**a**): The predicted mode of interaction of hGSTM1-1 with the insecticide pirimicarb. Side chains of residues in contact with the ligand (cut-off distance 3.0 Å) are shown with yellow lines. (**b**): Superposition of pirimicarb (yellow color) with the GSH and CDNB conjugate (GSH-CDNB, red color). (**c**): Electrostatic potential surface around pirimicarb. Red, blue, and white show negative (−5), positive (+5) and neutral (0.0) charges, respectively.

A molecular-docking study was performed to obtain a detailed picture of pirimicarb interactions with hGSTM1-1. Since co-crystallization of enzyme/pirimicarb was not achieved due to the high-salt condition in the crystallization medium, a molecular-docking study was carried out, taking advantage of the high-resolution structure reported here. Therefore, the molecular-docking study was carried out using the new crystal structure of hGSTM1-1. The most likely mode of interaction of the pirimicarb with hGSTM1-1 is shown in Figure 6. The compound binds to the enzyme at a distinct site that partially overlaps with the H-site. This site appears to be the same as that identified by Hearne and Colman (2005) as the monobromobimane (mBBr) binding site in rat GSTM1-1, which is localized to an area midway through α-helix 4 (residues 90–114) [40]. The mixed-type inhibition observed in the kinetic study can be adequately interpreted assuming this binding topology. Superposition of the binding site of pirimicarb and S-nitrobenzyl-GSH showed that pirimicarb overlaps with the CDNB at H-site since the aromatic ring is positioned to form hydrogen bonds with Tyr6, Trp7, Leu12, His107, Met108, Met112 and Tyr115. Additionally, the molecule packs in such a way that it forms van der Waals contacts with Phe208 and Ser209.

**Figure 7:**
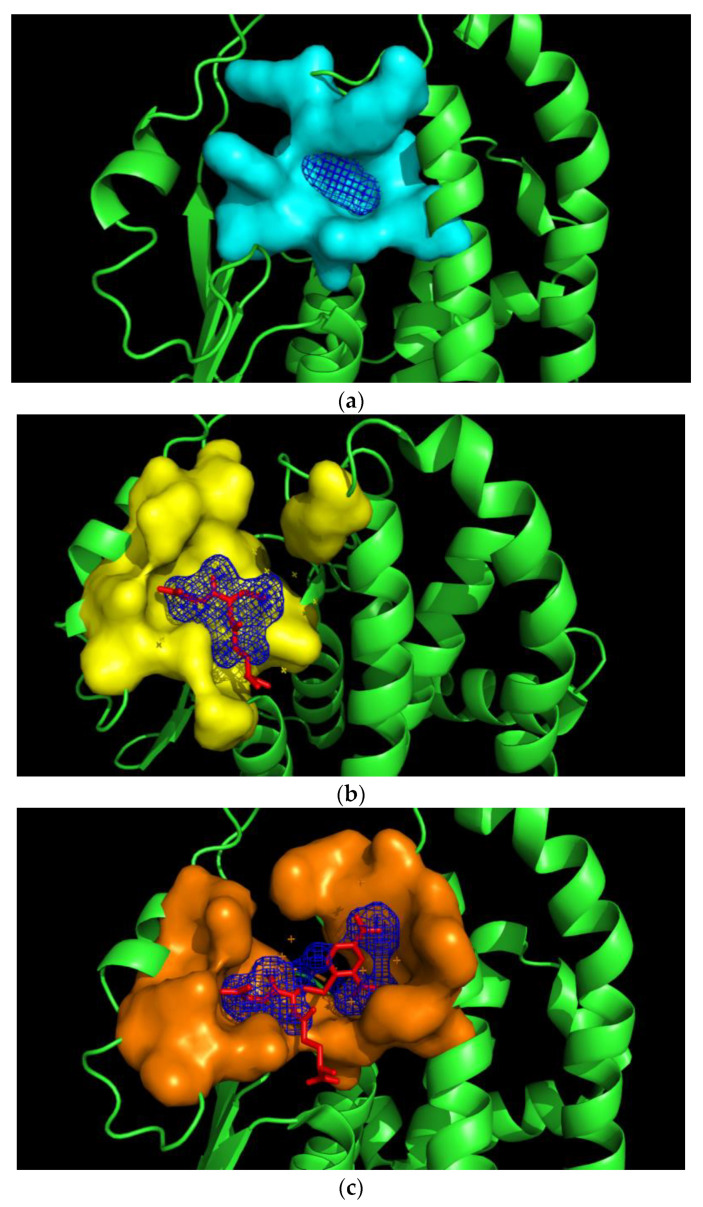
(**a**): The cavity volume of the ligand-free human GSTM1-1. (**b**): The cavity volume of the human GSTM1-1 in complex with GSH. (**c**): The cavity volume of the human GSTM1-1 in complex with GSH and CDNB. Figures have been created with KVFinder (PyMOL plug-in).

A crucial difference between the other structures of the hGSTM1-1 is the presence and the absence of GSH or S-nitrobenzyl-GSH. The comparison of the crystal structure resolved in the present study with the available GSH-bound hGSTM1-1 (1.9 Å resolution, PDB id: 1xw6) and the crystal structure of S-nitrobenzyl-GSH-bound hGSTM1-1 (2.3 Å resolution, PDB id: 1xwk) showed root-mean-square deviation values (RMSDs) of 0.28 Å and 0.65 Å, respectively. It is well-established that GSTs bind many structurally different molecules as a consequence of their structural plasticity and flexibility. For example, analysis of the three structures showed that the volume of the substrate-binding cavity is adapted to the size of the bound ligand. Indeed, the cavity volume for the ligand-free hGSTM1-1 is ~8.6 Å^3^, for the GSH-bound enzyme it is ~32.4 Å^3^ and for the GSH-CDNB conjugate the cavity volume is ~55.7 Å^3^ (Figure 7).

This is probably a consequence of the high plasticity and conformational flexibility of the binding site, such that in the absence of its natural substrate (e.g., GSH) the volume of the binding site shrinks and becomes less accessible to large ligands. Conformational fluctuations of GSTs can lead to ensembles of free enzyme, E, and both the ES and EP complexes. Since the binding of substrates and the ligand (e.g., pirimicarb) occur within the same active site, presumably, two simplified scenarios can explain the limiting promiscuity of hGSTM1-1 [33,42,43]. Assuming the classic induced-fit model, conformational changes occur only after binding of the substrate, where substrate binding leads to an ES complex with a different conformation to enzyme E. On the other hand, according to the conformational selection model, an ensemble of conformers pre-exists to allow for the binding of a substrate or ligand to a pre-organized enzyme structure suitable for that substrate or ligand. A population shift may take place as a consequence of the conformational selection mechanism. The conformational selection model may be exploited by GSTM1-1; different conformations in the ensemble would likely be selected by GSH, S-nitrobenzyl-GSH or pirimicarb and the ensemble could provide a mechanism to achieve selection and restricted ligandability.

## 3. Materials and Methods

### 3.1. Chemicals

Reduced glutathione (GSH), 1-chloro-2,4-dinitrobenzene (CDNB), bovine serum albumin (BSA), kanamycin, acetochlor, butachlor, metazachlor and propachlor were obtained from Sigma-Aldrich Co. (St. Louis, MO, USA). The pesticides alachlor and malathion were obtained from Fluka. The rest of the pesticides were purchased from Riedel de Haen (Seelze, Germany).

### 3.2. Heterologous Expression, Purification of hGSTM1-1 in E. coli and Protein Determination

Recombinant *E. coli* cells were grown in Luria–Bertani (LB) medium containing kanamycin (25 mg/mL) as described by Georgakis et al., 2017 [19]. Purification of hGSTM1-1 was carried out by affinity chromatography as described by Georgakis et al., 2017 [19]. Protein purity was >98% as determined by 12% SDS-PAGE. Protein concentrations were determined according to the Bradford method [44] using BSA as standard.

### 3.3. Assay of Enzyme Activity and Inhibition Analysis

Reduced GST assays were performed by monitoring the formation of the conjugate of CDNB (1 mM) with GSH (2.5 mM) at 340 nm (ε = 9.6 mM^−1^cm^−1^) for 120 s according to a published method [19]. One unit of enzyme activity is defined as the amount of enzyme that produces 1 μmol of product per minute under the assay conditions.

Pesticide screening and inhibition potency evaluation towards hGSTM1-1 were carried out in the assay system described above in the presence of 25 μM pesticide diluted in acetone. During the assay, no measurable pesticide/GSH reaction was detected. The percentage of enzyme inhibition (I) was calculated using the following equation:

%I = [(Ro − Ri)/Ro] × 100(3)

where R_o_ is the unit of enzyme activity increase for the uninhibited reaction and R_i_ is the rate of increase for the inhibited reaction. Both R_i_ and R_o_ correspond to the same substrate concentration.

The IC_50_ values were determined in the assay system described above by measuring the hGSTM1-1 activity in the presence of different concentrations of pirimicarb (0–240 μM), fenhexamid (0–500 μM) and butachlor (0–400 μM) at 37 °C. The IC_50_ values were determined by fitting the following equation to the concentration–response data:

%Inhibition = 100/[1 + (IC_50_/[I])] (4)

where [I] is the pesticide concentration (inhibitor). The IC_50_ values were determined using the program GraphPad Prism (GraphPad Software Inc., Version 7, San Diego, CA, USA).

### 3.4. Kinetic Inhibition Study with the Insecticide Pirimicarb

Initial velocities for the catalyzed reaction of the isoenzyme hGSTM1-1 with GSH as a variable substrate were performed at 37 °C in a total volume of 1 mL mixture containing 0.75 μg of the isoenzyme GSTM1-1, 0.1 M potassium phosphate buffer (pH 6.5), 1 mM CDNB and different concentrations of GSH in the range of 0.0175–3.75 mM in the presence of different concentrations of the insecticide pirimicarb (0–40 μM). With CDNB as a variable substrate, the reaction mixture total volume (1 mL) contained 0.75 μg of the isoenzyme GSTM1-1, 0.1 M potassium phosphate buffer (pH 6.5), 1 mM GSH and CDNB in the concentration range of 0.015–1.5 mM in the presence of different concentrations of pirimicarb (0–30 μM). The kinetic parameters were determined using the computer program GraphPad Prism 7.

### 3.5. Crystallization and Structure Determination

The protein was concentrated to 10 mg/mL in buffer HEPES pH 7.5, NaCl 150 mM, NaN_3_ 0.002% *w*/*v*. Crystals were produced with the hanging-drop vapor diffusion method using a reservoir solution of HEPES 0.1 m (pH 7.2), PEG 6000 18% (*w*/*v*) and ammonium chloride 0.2 M. X-ray diffraction data were collected on the P13 beamline at PETRA III (DESY, Hamburg, Germany) at cryogenic temperature (−173.1 °C) from crystals flash-cooled with liquid nitrogen in the presence of 20% (*v*/*v*) glycerol as cryoprotectant. A Dectris Pilatus 6M detector was used and the total exposure time was 120 sec for 3000 frames (0.04 s per frame). The data were processed with XDS [45] and AIMLESS [46]. The structure was solved by molecular replacement using the structure of the ligand-free hGSTM1-1 (PDB id 1gtu) [38] as a search model in PHASER [47]. Refinement was carried out with PHENIX v. 1.17.1-3660 [48] using simulated annealing and maximum likelihood as a target function. Structure visualization and rebuilding were carried out with Coot [49]. Structure validation was performed using Coot and PHENIX validation tools. Crystallographic data collection and final refinement statistics are given in Table 1.

### 3.6. Docking of Insecticide Pirimicarb into the hGSTM1-1 Binding Site

Molecular docking was carried out using the Autodock Vina tool of UCSF Chimera 1.11.2 [50]. Default parameters were employed for docking. The structure of the ligand-free hGSTM1-1, resolved and reported in the present study, was used as a receptor in the docking experiment.

### 3.7. Irreversible Inactivation of hGSTM1-1 by S-glutathionyl Ethylenesulfonyl Fluoride (GS-ESF)

Irreversible inactivation of hGSTM1-1 was performed in an incubation mixture containing, in 1 mL total volume (25 °C): 0.1 M KH_2_PO_4_, pH = 7.4, GS-ESF (0.025–0.2 mM) and hGSTM1-1 (approximately 0.1 U). The rate of inactivation was followed by periodically removing samples for assays of enzymatic activity. Initial rates of inactivation were deduced from plots of log (% of activity remaining) versus time (min) for several GS-ESF concentrations. Irreversible inactivation of hGSTM1-1 by GS-ESF was also carried out, in the presence of S-nitrobenzyl-glutathione (1 mM) or pirimicarb (15 μM), performed essentially as described above.

## 4. Conclusions

Determining GSTM1-1 ligandability remains a challenging task. Although several groups have pushed research forward to identify inhibitors against other classes of GSTs, such as GSTA1-1 and GSTP1-1, the isoenzyme GSTM1-1 appears to display restricted ligandability. This is probably a consequence of the high plasticity and conformational flexibility of the binding site, whose volume is reduced and becomes less accessible to large ligands in the absence of its natural substrate (e.g., GSH). The results of the present work allowed for the mapping of the xenobiotic ligand-binding properties of hGSTM1-1, and they form the basis for the design of new and selective lead compounds as inhibitors towards hGSTM1-1.

## Figures and Tables

**Table 1:** X-ray data collection and refinement statistics.

Wavelength (Å)	1.033
Resolution range	52.8–1.59 (1.65–1.59)
Space group	*P*2_1_
Unit cell	49.12 211.19 49.44 90 116.23 90
Total reflections	310250
Unique reflections	112127 (8772)
Multiplicity	2.8 (2.1)
Completeness (%)	93.36 (73.05)
Mean I/sigma(I)	10.6 (2.0)
Wilson B-factor (Å^2^)	20.74
R-meas	0.064 (0.466)
R-pim	0.045 (0.330)
CC_1/2_	1.0 (0.78)
Reflections used in refinement	112117 (8772)
Reflections used for R-free	5544 (495)
R-work/R-free	0.1983 (0.2844)/0.2358 (0.3190)
Number of non-hydrogen atoms	8454
macromolecules	7258
ligands (Na atoms)	7
solvent	1149
Protein residues	872
RMS in bonds (Å)	0.007
RMS in angles (°)	0.90
Ramachandran favored (%)	98.50
Ramachandran allowed (%)	1.39
Ramachandran outliers (%)	0.12
Rotamer outliers (%)	0.00
Clashscore	5.72
Average B-factor (Å^2^)	25.39
macromolecules	23.99
ligands	37.86
solvent	33.88
PDB id	7beu

## Data Availability

Data are contained within the article.

## References

[1] Townsend D.M., Tew K.D. (2003). The role of glutathione-S-transferase in anti-cancer drug resistance. Oncogene.

[2] Kumar G.N., Surapaneni S. (2001). Role of drug metabolism in drug discovery and development. Med. Res. Rev..

[3] Hayes J.D., Flanagan J.U., Jowsey I.R. (2005). Glutathione Transferases. Annu. Rev. Pharmacol. Toxicol..

[4] Kapoli P., Axarli I.A., Platis D., Fragoulaki M., Paine M., Hemingway J., Vontas J., Labrou N.E. (2008). Engineering sensitive glutathione transferase for the detection of xenobiotics. Biosens. Bioelectron..

[5] Chronopoulou E., Labrou N. (2009). Glutathione Transferases: Emerging Multidisciplinary Tools in Red and Green Biotechnology. Recent Pat. Biotechnol..

[6] Wu B., Dong D. (2012). Human cytosolic glutathione transferases: Structure, function, and drug discovery. Trends Pharmacol. Sci..

[7] Alves C.S., Kuhnert D.C., Sayed Y., Dirr H.W. (2006). The intersubunit lock-and-key motif in human glutathione transferase A1-1: Role of the key residues Met51 and Phe52 in function and dimer stability. Biochem. J..

[8] Sayed Y., Wallace L.A., Dirr H.W. (2000). The hydrophobic lock-and-key intersubunit motif of glutathione transferase A1-1: Implications for catalysis, ligandin function and stability. FEBS Lett..

[9] Rossjohn J., McKinstry W.J., Oakley A.J., Verger D., Flanagan J., Chelvanayagam G. (1998). Human theta class glutathione transferase: The crystal structure reveals a sulfate-binding pocket within a buried active site. Structure.

[10] Chronopoulou E., Kontouri K., Chantzikonstantinou M., Pouliou F., Perperopoulou F., Voulgari G., Bosmali E., Axarli I., Nianiou-Obeidat I., Madesis P. (2014). Plant glutathione transferases: Structure, antioxidant catalytic function and in planta protective role in biotic and abiotic stress. Curr. Chem. Biol..

[11] Pljesa-Ercegovac M., Savic-Radojevic A., Matic M., Coric V., Djukic T., Radic T., Simic T. (2018). Glutathione Transferases: Potential Targets to Overcome Chemoresistance in Solid Tumors. Int. J. Mol. Sci..

[12] McIlwain C.C., Townsend D.M., Tew K.D. (2006). Glutathione S-transferase polymorphisms: Cancer incidence and therapy. Oncogene.

[13] Albarakati N., Khayyat D., Dallol A., Al-Maghrabi J., Nedjadi T. (2019). The prognostic impact of GSTM1/GSTP1 genetic variants in bladder cancer. BMC Cancer.

[14] Tew K.D., Manevich Y., Grek C., Xiong Y., Uys J., Townsend D.M. (2011). The role of glutathione S-transferase P in signaling pathways and S-glutathionylation in cancer. Free Radic. Biol. Med..

[15] Economopoulos K.P., Sergentanis T.N. (2010). GSTM1, GSTT1, GSTP1, GSTA1 and colorectal cancer risk: A comprehensive meta-analysis. Eur. J. Cancer.

[16] Federici L., Sterzo C.L., Pezzola S., Di Matteo A., Scaloni F., Federici G., Caccuri A.M. (2009). Structural basis for the binding of the anticancer compound 6-(7-Nitro-2,1,3-Benzoxadiazol-4-Ylthio)hexanol to human glutathione S-transferases. Cancer Res..

[17] Wang M., Li Y., Lin L., Song G., Deng T. (2016). GSTM1 Null Genotype and GSTP1 Ile105Val Polymorphism Are Associated with Alzheimer’s Disease: A Meta-Analysis. Mol. Neurobiol..

[18] Jafarian Z., Saliminejad K., Kamali K., Ohadi M., Kowsari A., Nasehi L., Khorram H.R. (2018). Association of glutathione S-transferases M1, P1 and T1 variations and risk of late-onset Alzheimer’s disease. Neurol. Res..

[19] Georgakis N.D., Karagiannopoulos D.A., Thireou T.N., Eliopoulos E.E., Labrou N.E., Tsoungas P.G., Koutsilieris M.N., Clonis Y.D. (2017). Concluding the trilogy: The interaction of 2, 2′-dihydroxy-benzophenones and their carbonyl N-analogues with human glutathione transferase M1-1 face to face with the P1-1 and A1-1 isoenzymes involved in MDR. Chem. Biol. Drug. Des..

[20] Board P., Menon D. (2013). Glutathione transferases, regulators of cellular metabolism and physiology. Biochim. Biophys. Acta. Bioenerg..

[21] Uppugunduri C.R.S., Muthukumaran J., Robin S., Santos-Silva T., Ansari M. (2022). In silico and in vitro investigations on the protein-protein interactions of glutathione S-transferases with mitogen-activated protein kinase 8 and apoptosis signal-regulating kinase 1. J. Biomol. Struct. Dyn..

[22] Robin S., Ben Hassine K., Mlakar S.J., Mlakar V., Ansari M., Uppugunduri C.R.S. (2021). The Catalytic Activity of GSTM1 In vitro is Independent of MAPK8. Drug Metab. Lett..

[23] Schultz M., Dutta S., Tew K.D. (1997). Inhibitors of glutathione S-transferases as therapeutic agents. Adv. Drug Deliv. Rev..

[24] Duvoix A., Morceau F., Delhalle S., Schmitz M., Schnekenburger M., Galteau M.-M., Dicato M., Diederich M. (2003). Induction of apoptosis by curcumin: Mediation by glutathione S-transferase P1-1 inhibition. Biochem. Pharmacol..

[25] Alqarni M.H., Foudah A.I., Muharram M.M., Labrou N.E. (2021). The Interaction of the Flavonoid Fisetin with Human Glutathione Transferase A1-1. Metabolites.

[26] Kulaksiz-Erkmen G., Dalmizrak O., Dincsoy-Tuna G., Dogan A., Ogus I.H., Ozer N. (2011). Amitriptyline may have a supportive role in cancer treatment by inhibiting glutathione S-transferase pi (GST-π) and alpha (GST-α). J. Enzym. Inhib. Med. Chem..

[27] Luisi G., Mollica A., Carradori S., Lenoci A., De Luca A., Caccuri A.M. (2016). Nitrobenzoxadiazole-based GSTP1-1 inhibitors containing the full peptidyl moiety of (pseudo) glutathione. Enzyme Inhib Med Chem..

[28] Shishido Y., Tomoike F., Kuwata K., Fujikawa H., Sekido Y., Murakami-Tonami Y. (2019). A Covalent Inhibitor for Glutathione S-Transferase Pi (GSTP1-1) in Human Cells. ChemBioChem.

[29] Pushpakom S., Iorio F., Eyers P.A., Escott K.J., Hopper S., Wells A., Doig A., Guilliams T., Latimer J., McNamee C. (2019). Drug repurposing: Progress, challenges and recommendations. Nat. Rev. Drug Discov..

[30] Pouliou F.M., Thireou T.N., Eliopoulos E.E., Tsoungas P.G., Labrou N.E., Clonis Y.D. (2015). Isoenzyme-and Allozyme-Specific Inhibitors: 2, 2′-Dihydroxybenzophenones and Their Carbonyl N-Analogues that Discriminate between Human Glutathione Transferase A1-1 and P1-1 Allozymes. Chem. Biol. Drug Des..

[31] Perperopoulou F.D., Tsoungas P.G., Thireou T.N., Rinotas V.E., Douni E.K., Eliopoulos E.E., Labrou N.E., Clonis Y.D. (2014). 2, 2′-Dihydroxybenzophenones and their carbonyl N-analogues as inhibitor scaffolds for MDR-involved human glutathione transferase isoenzyme A1-1. Bioorg. Med. Chem..

[32] Perperopoulou F., Ataya F.S., Fouad D., Malik A., Saeed H.M., Labrou N.E. (2016). Biochemical characterization of the detoxifying enzyme glutathione transferase P1-1 from the camel Camelus dromedarius. Cell Biochem. Biophys..

[33] Bauer U., Breeze A.L. (2016). “Ligandability” of drug targets: Assessment of chemical tractability via experimental and *in silico* approaches. Lead Generation.

[34] Kitz R., Wilson I.B. (1962). Esters of methanesulfonic acid as irreversible inhibitors of acetylcholinesterase. J. Biol. Chem..

[35] Ralat L.A., Manevich Y., Fisher A.B., Colman R.F. (2006). Direct evidence for the formation of a complex between 1-cysteine peroxiredoxin and glutathione S-transferase π with activity changes in both enzymes. Biochemistry.

[36] Alqarni M.H., Foudah A.I., Muharram M.M., Labrou N.E. (2021). The Interaction of Human Glutathione Transferase GSTA1-1 with Reactive Dyes. Molecules.

[37] Platis M., Vlachakis D., Foudah A.I., Muharram M.M., Alqarni M.H., Papageorgiou A.C., Labrou N.E. (2021). The interaction of *Schistosoma japonicum* glutathione transferase with Cibacron blue 3GA and its fragments. J. Med. Chem..

[38] Patskovsky Y.V., Patskovska L.N., Listowsky I. (1999). Functions of His107 in the catalytic mechanism of human glutathione S-transferase hGSTM1a-1a. Biochemistry.

[39] Chern M.K., Wu T.C., Hsieh C.H., Chou C.C., Liu L.F., Kuan I.C. (2000). Tyr115, gln165 and trp209 contribute to the 1, 2-epoxy-3-(p-nitrophenoxy) propane-conjugating activity of glutathione S-transferaSE CgSTM1-1. J. Mol. Biol..

[40] Hearne J.L., Colman R.F. (2005). Delineation of xenobiotic substrate sites in rat glutathione S-transferase M1-1. Protein Sci..

[41] Shishido Y., Tomoike F., Kimura Y., Kuwata K., Yano T., Fukui K., Fujikawa H., Sekido Y., Murakami-Tonami Y., Kameda T. (2017). A covalent G-site inhibitor for glutathione S-transferase Pi (GSTP_1-1_). Chem. Commun..

[42] Surade S., Blundell T.L. (2012). Structural biology and drug discovery of difficult targets: The limits of ligandability. Chem. Biol..

[43] Dang C.V., Reddy E.P., Shokat K.M., Soucek L. (2017). Drugging the undruggable cancer targets. Nat. Rev. Cancer.

[44] Bradford M.M. (1976). A rapid and sensitive method for the quantification of microgram quantities of protein utilizing the principle of protein-dye binding. Anal. Biochem..

[45] Kabsch W. (2010). Xds. Acta Crystallogr. Sect. D Biol. Crystallogr..

[46] Evans P.R., Murshudov G.N. (2013). How good are my data and what is the resolution?. Acta Crystallogr. Sect. D Biol. Crystallogr..

[47] McCoy A.J., Grosse-Kunstleve R.W., Adams P.D., Winn M.D., Storoni L.C., Read R.J. (2007). Phaser crystallographic software. J. Appl. Crystallogr..

[48] Liebschner D., Afonine P.V., Baker M.L., Bunkóczi G., Chen V.B., Croll T.I. (2019). Macromolecular structure determination using X-rays, neutrons and electrons: Recent developments in Phenix. Acta Crystallogr. Sect. D Struct. Biol..

[49] Emsley P., Cowtan K. (2004). Coot: Model-building tools for molecular graphics. Acta Crystallogr. Sect. D Biol. Crystallogr..

[50] Butt S.S., Badshah Y., Shabbir M., Rafiq M. (2020). Molecular Docking Using Chimera and Autodock Vina Software for Nonbioinformaticians. Bioinform. Biotechnol..

